# Genome-Wide Association Mapping of Mixed Linkage (1,3;1,4)-β-Glucan and Starch Contents in Rice Whole Grain

**DOI:** 10.3389/fpls.2021.665745

**Published:** 2021-08-25

**Authors:** Rahele Panahabadi, Asadollah Ahmadikhah, Lauren S. McKee, Pär K. Ingvarsson, Naser Farrokhi

**Affiliations:** ^1^Department of Plant Science and Biotechnology, Faculty of Life Sciences and Biotechnology, Shahid Beheshti University, Tehran, Iran; ^2^Division of Glycoscience, Department of Chemistry, KTH Royal Institute of Technology, AlbaNova University Centre, Stockholm, Sweden; ^3^Wallenberg Wood Science Centre, Stockholm, Sweden; ^4^Linnean Centre for Plant Biology, Department of Plant Biology, Swedish University of Agricultural Sciences, Uppsala, Sweden

**Keywords:** candidate gene, GWAS, MLG, *Oryza sativa*, SNP, starch

## Abstract

The glucan content of rice is a key factor defining its nutritional and economic value. Starch and its derivatives have many industrial applications such as in fuel and material production. Non-starch glucans such as (1,3;1,4)-β-D-glucan (mixed-linkage β-glucan, MLG) have many benefits in human health, including lowering cholesterol, boosting the immune system, and modulating the gut microbiome. In this study, the genetic variability of MLG and starch contents were analyzed in rice (*Oryza sativa* L.) whole grain, by performing a new quantitative analysis of the polysaccharide content of rice grains. The 197 rice accessions investigated had an average MLG content of 252 μg/mg, which was negatively correlated with the grain starch content. A new genome-wide association study revealed seven significant quantitative trait loci (QTLs) associated with the MLG content and two QTLs associated with the starch content in rice whole grain. Novel genes associated with the MLG content were a hexose transporter and anthocyanidin 5,3-*O*-glucosyltransferase. Also, the novel gene associated with the starch content was a nodulin-like domain. The data pave the way for a better understanding of the genes involved in determining both MLG and starch contents in rice grains and should facilitate future plant breeding programs.

## Introduction

Rice (*Oryza sativa* L.) improvement for increased nutritional value is a crucial objective in the breeding programs. Much of the nutritious value of the rice grain derives from carbohydrates found in the plant cell wall and those used for energy storage within the grain. Plant cell walls are composites of polymer chains mainly derived from monosaccharides and phenolic compounds, with cellulose and lignin acting as the strong fibrous components surrounded by more amorphous matrix polysaccharides. The latter are often referred to as hemicelluloses and include glucuronoarabinoxylans (GAXs), arabinoxylans (AXs), mixed linkage (1,3;1,4)-β-D-glucans (MLGs), xyloglucans (XyGs), and galactoglucomannans (GGMs). The hemicelluloses may be embedded in a gel of pectic polysaccharides, depending on the plant species (Burton et al., 2010).

**Figure 1:**
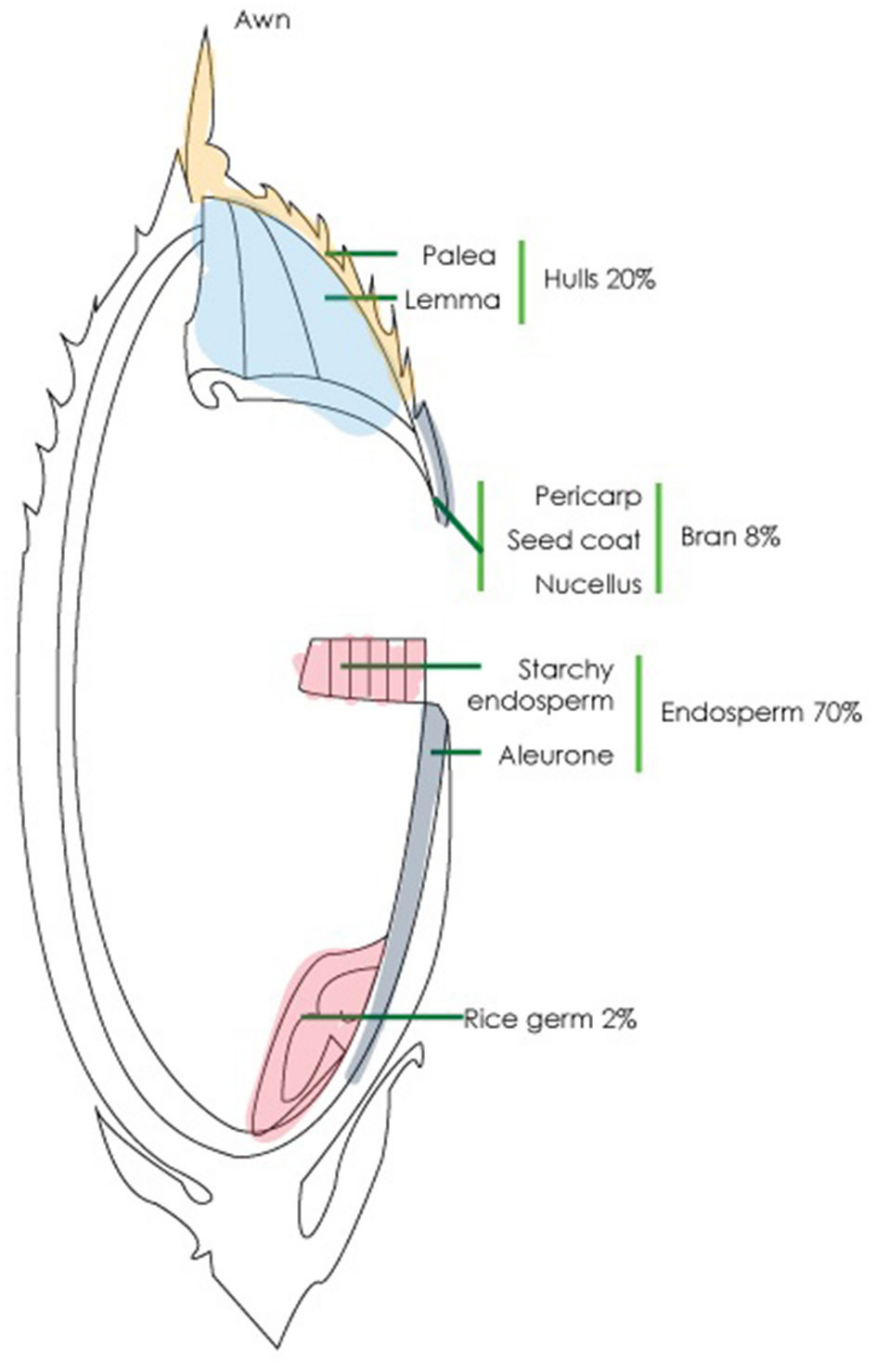
Structure of rice whole grain. The grain consists of four main parts, namely, hulls (20%), bran (8%), endosperm (70%), and germ (2%).

As the carbohydrate content of rice grains, which may be consumed with or without an intact bran layer, is so vital to any assessment of the nutritional value of new breeds of rice, we determined to study the content of some key polysaccharides within a panel of rice accessions. Rice endosperm cells, similar to other members of the *Poaceae*, contain primary cell walls rich with AXs, but otherwise, the major dietary polysaccharides of rice are the α- and β-glucans. Rice endosperm contains up to 70% starch (Figure 1), which is used for energy storage and is not found in the cell wall itself (Moongngarm, 2013). Rice bran is a by-product of the milling industry as it is most often removed before the rice is sold for human consumption; the bran constitutes ~8% of the initial grain weight and is comprised of ~80% of carbohydrates (Gul et al., 2015) that mostly consist of cellulose, matrix polysaccharides, and pectin (Saunders, 1990). AXs and MLGs constitute about 4.8–8.5% and 6% of the rice bran, respectively (Hashimoto et al., 1987; Sharma et al., 2004; Collins et al., 2010). The variability of MLG and starch contents in the whole grain of different rice accessions has not previously been studied in detail.

**Figure 2:**
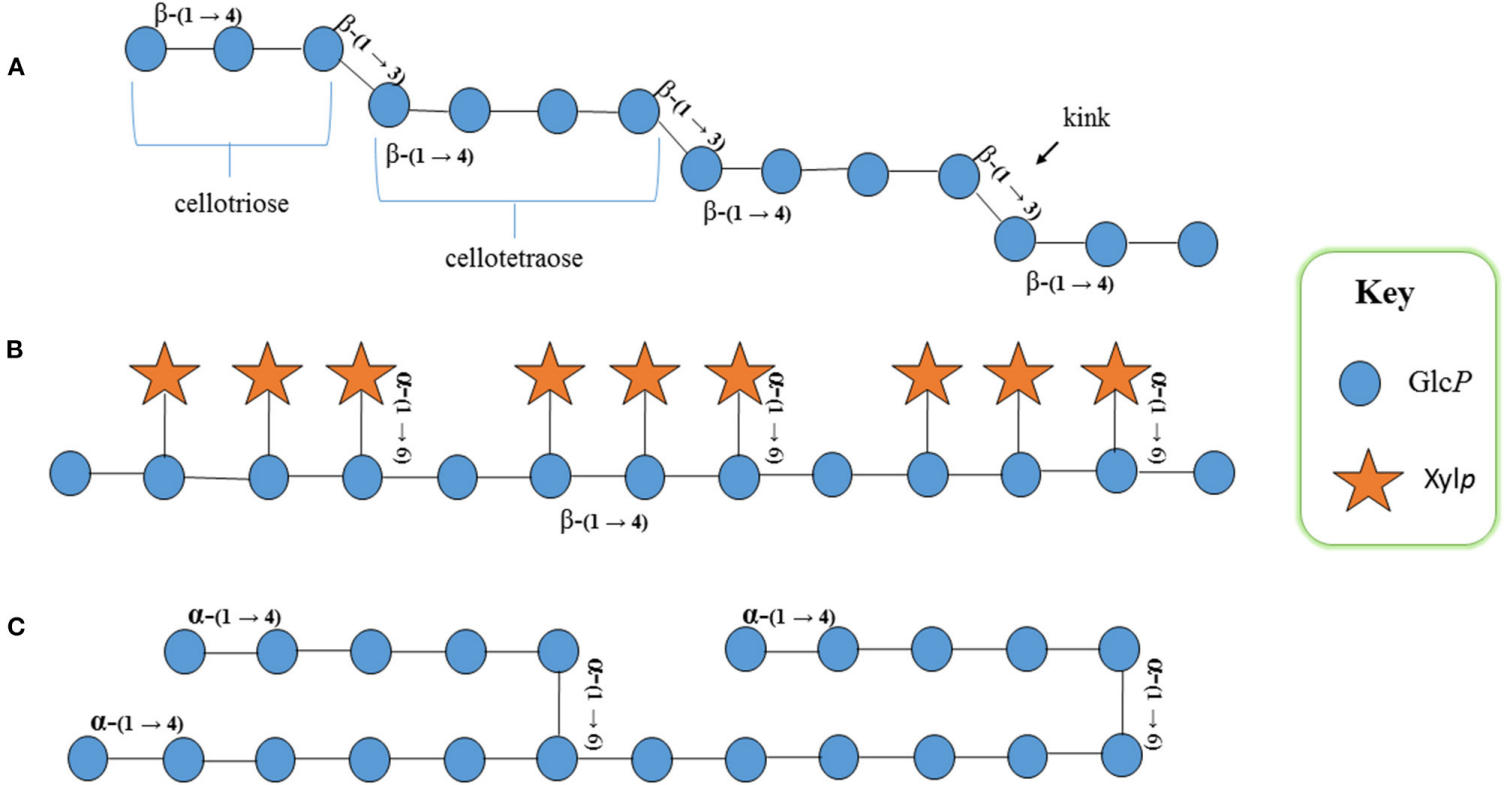
Schematic structural representation of rice **(A)** mixed linkage (1,3;1,4)-β-D-glucan (MLG): cellotriose and cellotetraose with β-(1→4) linkages are interconnected with β-(1→3) linkages that produce a kink in the structure, conveying properties different to other glucans in the cell wall, **(B)** xyloglucan: β-(1→4)-d-glucan backbone substituted with α-(1→6) xylosyl residues; the fourth glycosyl residue on the backbone remains unsubstituted, and **(C)** starch: α-(1→4)-d-glucan backbone that remains unbranched in amylose and branched in amylopectin. Glc*P*, glucopyranosyl residue; Xyl*P*, xylopyranosyl residue.

MLGs are linear β-(1→4)-D-glucans with irregularly spaced β-(1→3)-D-glucosyl residues in the main chain (Figure 2A). They represent a significant proportion of the total dietary fiber intake of many human diets. Their roles in plants include structural and storage properties in grain and other tissues. MLGs offer many benefits to human health, including promoting the production of beneficial short-chain fatty acids (SCFAs) from dietary fiber (Beta and Camire, 2019), reduction of colorectal cancer risk (Ferguson and Harris, 2003), immunostimulation of leukocytes (Yun et al., 2003), reduction of the glycemic index (Hodge et al., 2004), facilitation of bowel movement (Spagnuolo et al., 2017), and overall promotion of high-density lipoprotein (HDL) and reduction of low-density lipoprotein (LDL) (Martin et al., 2012). Antioxidant properties of MLGs have also been reported, leading to their use in cosmetic products, as they may stimulate elastin production in the skin (Kivela et al., 2011). Despite these many health benefits for humans, grains with high MLG content are considered to have lower feeding quality for domestic ruminant animals (Jacob and Pescatore, 2014). Additionally, high amounts of MLGs increase the viscosity of malt extracts, rendering them unsuitable in brewing industries (Fincher, 2009a). Hence, the MLG content of a range of rice grains should be profiled to allow the use of the optimal variety in each case.

The original discovery of genes involved in the biosynthesis of MLGs stems from early observations that dicots lack the polysaccharide (Carpita and Gibeaut, 1993). With the discovery of cellulose synthases (*CesAs*) in cotton (Pear et al., 1996), homology studies revealed the presence of paralogous sequences to these genes with no functional relevance to cellulose biosynthesis (Schwerdt et al., 2015). The term *cellulose synthase-like* (*CSL*) genes was coined, and the differential analysis of genome sequences for *CSLs* in rice and *Arabidopsis* at the time defined subfamilies and differences in terms of gene presence/absence between monocots and dicots (Hazen et al., 2002). MLG is found in cereals with *CSLs* from monocot-specific families, namely *CSLFs* (Hazen et al., 2002), *CSLHs* (Hazen et al., 2002), and *CSLJs* (Farrokhi et al., 2006; Fincher, 2009b; Little et al., 2018). Burton et al. (2006) and Doblin et al. (2009) expressed barley *CSLF* and *CSLH* genes, respectively, in *Arabidopsis* to make gain-of-function mutants. The experiments resulted in the accumulation of MLGs, supporting the hypothesis that these subfamilies contain MLG synthases. Heterologous expression of a *CSLJ* gene recently led to the biosynthesis of MLGs by recombinant enzyme, confirmed by immunocytochemical analyses (Little et al., 2018). To the best of our knowledge, there is as yet no definitive report on MLG-modifying enzymes or regulatory elements controlling the level of MLG synthesis *in planta*.

Xyloglucans, highly branched polysaccharides of the primary wall interconnecting with cellulose microfibrils *via* hydrogen bonds and found in all land plants, have a glucosyl backbone substituted at *O*-6 with xylopyranosyl (α-D-Xylp) residues (Figure 2B; Kato et al., 1982). Detailed studies of XyG biosynthesis and its cellular modification have revealed the participation of numerous enzymes, namely, β-(1→4)-glucan synthase (CslC) (Reiter et al., 1997; Dwivany et al., 2009; Kim et al., 2020), xyloglucan fucosyltransferase (XG FUT) (Perrin et al., 1999; Zabotina, 2012), xyloglucan galactosyltransferase (XG GalT) (Madson et al., 2003; Del Bem and Vincentz, 2010), xyloglucan 6-xylosyltransferase (XG XYLT) (Culbertson et al., 2016; Pauly and Keegstra, 2016), and xyloglucan transglycosylase/hydrolase (XTH) (de la Torre et al., 2002). XTHs have both xyloglucan *endo*-transglucosylase and *endo*-hydrolase activity that catalyze XyGs (Yokoyama et al., 2004).

Starch is a highly abundant and important component in rice grains, and the starch content is negatively correlated with that of MLG (Marcotuli et al., 2016). Starch is a polymer of glucosyl residues (Figure 2C), and the amount of starch present within the rice grain is influenced by the enzymes, namely, starch synthase, starch branching enzyme (SBE), ADP-glucose pyrophosphorylase, and starch de-branching enzyme (Qu et al., 2018). SBE generates amylopectin by cleaving internal amylose α-(1,4) glycosidic linkages and transferring the reducing ends to *C6* hydroxyls to form α-(1,6) linkages (Tetlow and Emes, 2014).

Genome-wide association studies (GWAS) and the availability of the genome sequences of crops have revolutionized our path toward gene discovery in the last two decades or so. For instance, GWAS for MLGs has been performed in oat (Asoro et al., 2013), barley (Houston et al., 2014), and durum wheat (Marcotuli et al., 2016), but the corresponding GWAS data for rice is missing. Despite the scarcity of reports of GWAS on β-glucans, data are available for starch as the dominant polysaccharide in cereal grains (Xu et al., 2018; Biselli et al., 2019; Chen et al., 2019). An understanding of the natural variability of β-glucans and starch in rice and their association with particular genetic loci could provide markers to enhance the nutritional value and industrial applications of specific rice accessions. In this study, the main goals were to quantify MLG and starch in a rice population (named association panel) and then to perform a GWAS to identify the genomic regions affecting the variability of MLG and starch contents in an international rice population, with a view to identifying accessions with particular relevance for food or other production industries.

## Materials and Methods

### Materials

Glucose, sodium azide, and myo-inositol were purchased from Sigma Aldrich (Berlin, Germany). Trifluoroacetic acid (TFA) was purchased from Merck (Berlin, Germany). A solution of 50% of NaOH used for high-performance liquid chromatography with pulsed amperometric detection (HPAEC-PAD) was purchased from Fisher Scientific (Berlin, Germany). The Total Starch Assay Kit (AA/AMG) was purchased from Megazyme (Bray, Ireland). All other chemicals were of the highest available chemical grade and were obtained from Merck.

### Plant Materials and Growth Conditions

In this study, we used 197 rice accessions; the associated file Supplementary Table 1 describes the accession number, common name, subpopulation, and country of origin for all accessions under investigation. The seeds for these accessions were obtained from the International Rice Research Institute (IRRI), Philippines. The seeds were planted in plots of 2 × 2 m^2^ with 25 cm spacing within rows at the Sari Agricultural University, Iran during the 2017–2019 cultivation season. The accessions used consisted of a mixture of landraces, breeding lines, varieties, and cultivars. The genotypes were evaluated using a randomized complete design with three replications. Fertilization of the trial was done using superphosphate triple at the plowing stage, urea at the seedling stage, and potash at the plowing stage, given to plants at 180:100:80 kg/ha.

The accessions belong to the following subpopulations (as defined by Zhao et al., 2011): TEJ (Temperate Japonica), IND (Indica), AUS (aus), ARO (Aromatic), TRJ (Tropical Japonica), and ADMIX. The data from the rice 44.1K SNP (Single Nucleotide Polymorphism) array were downloaded for all accessions from the Gramene portal (http://gramene.org). Single seed descent was performed on all genotypes in the subsequent year that followed with phenotyping. Plots were hand-harvested at maturity, and grain was stored at 4°C. Using a sample miller, the grain was ground and passed across a 0.1-μm sieve, and the flour samples were stored in the cold room.

### Genotyping by Sequencing and Imputation Method

The development and sequencing of SNP array hybridization for the rice population have previously been described by Zhao et al. (2011). In brief, 44,100 SNPs from two data sources, i.e., the OryzaSNP project and the OMAP project, were used for GWAS. In the designed experiments, BioPrime DNA Labeling Kit (Invitrogen, Cat. No: 18094-011) was used to generate the probe that is used for hybridization purposes according to the Affymetrix SNP 6.0 protocol.

### Quantification of Starch and MLG Contents

The MLG content was measured indirectly by subtracting the amount of starch in samples from the total noncellulosic glucose content. First, noncrystalline glucans (i.e., starch and MLG, but not cellulose) were quantified by hydrolyzing biomass using TFA as described by Zhang et al. (2012). The powdered grain sample (1 mg) was incubated with 1 ml of 2 M TFA for 3 h at 120°C. The released monosaccharides were resolved using a Dionex ICS 3000 HPAEC-PAD operated by using Chromeleon software version 6.80 (Dionex) using a Dionex CarboPac PA1 column. Solvent A was water, solvent B was 1 M sodium hydroxide, solvent C was 200 mM sodium hydroxide with 170 mM sodium acetate, and solvent D was 1 M sodium acetate. For the detection of glucose monosaccharides, the following gradient was employed: prewash and column calibration, 5–10 min 15% B (0.5 ml/min); sample injection, 0–16 min 15% B (0.5 ml/min); gradient elution, 15–30 min 33% B (0.5 ml/min), 30–31 min 33% B and 50% D (0.5 ml/min); column wash and final elution, 31–35 min 15% B (0.5 ml/min). The amount of glucose present in a sample was identified and quantified by comparison with the retention times and peak areas of neutral glucose standards applied at known concentrations. Then, the starch content of samples was measured using the Total Starch Assay Kit according to the instructions of the manufacturer, except that the method was scaled down from 100 mg of grains per sample to 1 mg of grains per sample. The amounts of noncellulosic β-glucans in samples were calculated by subtracting the determined starch content from the total glucose content previously determined by using TFA hydrolysis and HPAEC-PAD. The assumption was made that XyG (the other potential source of noncellulosic glucose) represents a very minor component of total glucose and could, therefore, be discounted from this calculation.

### Statistical Analysis of Phenotypic Data

Frequency distributions of phenotypes and the correlation between traits were plotted using the R package of programs (https://cran.r-project.org/). Normality of frequency distribution of phenotypic data was tested using Skewness and Kurtosis tests. MLG and starch contents were compared in different populations to determine significant differences between ecotypes. Broad-sense heritability ($H_{\text{b}}^{2}$) of the study traits was estimated using the Gaussian model implemented in the rptR package (https://cran.r-project.org/web/packages/rpart/index.html). The rptR package was used to estimate the heritability and phenotypic data with two replicates as recalled by R. The R package geneSLOPE (Brzyski et al., 2017) was used to calculate a specific false discovery rate (FDR), which considers intra-cluster correlations of SNPs (Dabney et al., 2010); the method implemented in the package is based on controlling the FDR of interesting discoveries by selecting hypotheses.

### Population Structure

Analyses of the population structure among all rice accessions were performed using the programs STRUCTURE (v2.2, Falush et al., 2007) using 36,902 SNP markers and TASSEL (v. 5) for the principal components analysis (PCA) (Bradbury et al., 2007). In the STRUCTURE analysis, the number of initially assumed subpopulations (*K*) was varied between 1 and 10. To increase the accuracy of the estimation, 10 independent replicates were specified for each *K* subpopulation. Burn-in was 100,000, Markov Chain Monte Carlo (MCMC) iterations were 100,000, and each *K* estimation was repeated 10 times. Finally, the most likely numbers of subpopulations (*K*) were estimated using the Δ*K* method of Evanno et al. (2005). The PCA analysis and the corresponding plot were generated using the genomic association and prediction integrated tool (GAPIT) (Lipka et al., 2012). The relationship between the accessions was visualized using a neighbor-joining dendrogram based on the pairwise distance matrix calculated using TASSEL v.5 and visualized using Archaeopteryx (Tang et al., 2016).

### Estimation of Linkage Disequilibrium (LD) Decay in Rice

Genome-wide linkage disequilibrium analyses were conducted among all 197 accessions in the association panel to evaluate the resolution of LD by performing pairwise calculations of LD between SNPs using *r*^2^ in a sliding window of 50 markers using TASSEL. Graphs depicting the decay of LD with the physical distance between SNPs were visualized using ggplot2 in R.

### Genome-Wide Association Mapping (GWAS)

The most suitable model was selected to obtain a higher level of reliance on the association results. Fixed and Random Model Circulating Probability Unification (FarmCPU) with three principal components (PCs), a recently developed model with fixed and random effects used to control false positives (Liu et al., 2016), was used for GWAS. TASSEL with two models [i.e., mixed linear model (MLM) and general linear model (GLM)] and GAPIT with three models [i.e., MLM, multiple loci mixed linear model (MLMM), and FarmCPU] were analyzed. The results from both TASSEL and GAPIT were evaluated based on the significance of associated loci using *t*-tests. Association analyses were performed using genotyping (out of 33,182 SNPs) and phenotyping data obtained from the accessions at GAPIT by using the FarmCPU (Liu et al., 2016). A Manhattan plot was generated using the –log_10_(*p*) values for every SNP with a 1% Bonferroni test threshold (Team, 2014). Markers with –log_10_(*p*) > 3.5 were considered as significant markers associated with MLG and starch contents. Despite the criterion of –log_10_(*p*) > 3.5, we considered a more stringent significance method known as FDRs, for selecting more reliable associations and candidate genes. The quantile–quantile (Q–Q) plot to determine which of the tested methods would be the best fit for the data. The Q–Q plots were generated by projecting observed vs. expected –log_10_*p* (Chen et al., 2016).

### Candidate Gene Analysis

To identify genes underlying the quantitative trait loci (QTLs) of the monosaccharide content that overlapped with the genomic regions (i.e., their associated SNPs), genes deposited on the Rice Annotation Project Database (http://rice.plantbiology.msu.edu/) and also IRGSP-1.0 (https://rapdb.dna.affrc.go.jp/) were assessed. All hypothetical genes were ignored to put protein-coding sequences and transposable elements into a prospect for further analyses. Flanking genes in 50 kb in either direction from any SNP marker (i.e., a total of 100 kb) were chosen for map order uncertainty and LD. This surrounding window of 100 kb was chosen due to the very slow LD decay occurring in rice (Mather et al., 2007). To ascertain whether candidate genes underlying our QTLs had been cloned, a thorough literature survey was carried out. BLASTn for expressed proteins was performed for the identified genes against *O. sativa* Japonica Group, *O. sativa* Indica Group, and *Hordeum vulgare* genomes at the Gramene portal (http://ensembl.gramene.org/) to determine the reported function.

The expression level of the candidate genes was obtained from the RGAP database (http://rice.plantbiology.msu.edu/), in which the expression was determined by two assays including RNA-Seq and TRAP-Seq. The expression level in different tissues (i.e., seedling, callus, and panicle) was reported based on Fragments Per Kilobase of transcript per Million mapped reads (FPKM). In addition, co-expression analyses for all the candidate genes were carried out using Genevestigator (https://genevestigator.com/) and RiceFrend (https://ricefrend.dna.affrc.go.jp/); the latter was used for the gene network and interaction analysis of the candidate genes. Furthermore, for a better understanding of the function of candidate genes in the cell, KEGG (Kyoto Encyclopedia of Genes and Genomes website) (https://www.kegg.jp/) and PANTHER (Protein Analysis Through Evolutionary Relationships website) (http://pantherdb.org/) were used.

## Results

### Phenotypic Traits

Analyses of traits, with the normal distribution (Supplementary Figure 1), showed large variations between the 197 rice accessions investigated (*p* < 0.001) (Table 1). The results showed that the amount of MLG varied in different rice genotypes and showed an average of 220 μg/mg of whole grain rice, and the amount of this carbohydrate in rice whole grain has not yet been measured. Extensive analyses of carbohydrate contents of rice grains found a negative correlation between the amount of MLG and starch; a high starch content is matched in rice grains with a low MLG content and *vice versa* (Supplementary Figure 2). The $H_{\text{b}}^{2}$ was 0.87 for MLG and 0.997 for starch (Table 1), indicating that the traits are largely under the control of genotypes with a few genes being involved in defining the cell wall polysaccharide contents in whole grain; the environment has a minimal effect. The rice accession panel was classified into six subpopulations using genotyping-by-sequencing (113,739 SNPs) according to Kim et al. (2016), and their distributions are presented in Supplementary Figure 3.

**Table 1:** Descriptive statistics for mixed linkage (1,3;1,4)-β-glucan (MLG) and starch content.

**Statistics**	**(1,3;1,4)-β-glucan (μg/mg)**	**Starch (μg/mg)**
Average	252	561
Minimum	27	321
Maximum	398	869
H^2^	0.875	0.997
df	196	196
Standard deviation(SD)	93.6040	118.6637
Coefficient of variation (C.V.) (%)	37.1	21.2

### SNP Data Curation

The total size of the rice genome is ~380 Mbp (Sasaki, 2005). On average, one SNP per 11.2 Kbp was found across all 12 rice chromosomes. The highest and the lowest numbers of SNPs were found on chromosome 1 (6,466 SNPs), and chromosome 10 (1,719 SNPs), respectively. Following the elimination of monomorphic loci and loci with minor allele frequency (MAF <0.05), the numbers of remaining markers were 33,812 from the total of 44,100 SNP markers.

### Population Structure

**Figure 3:**
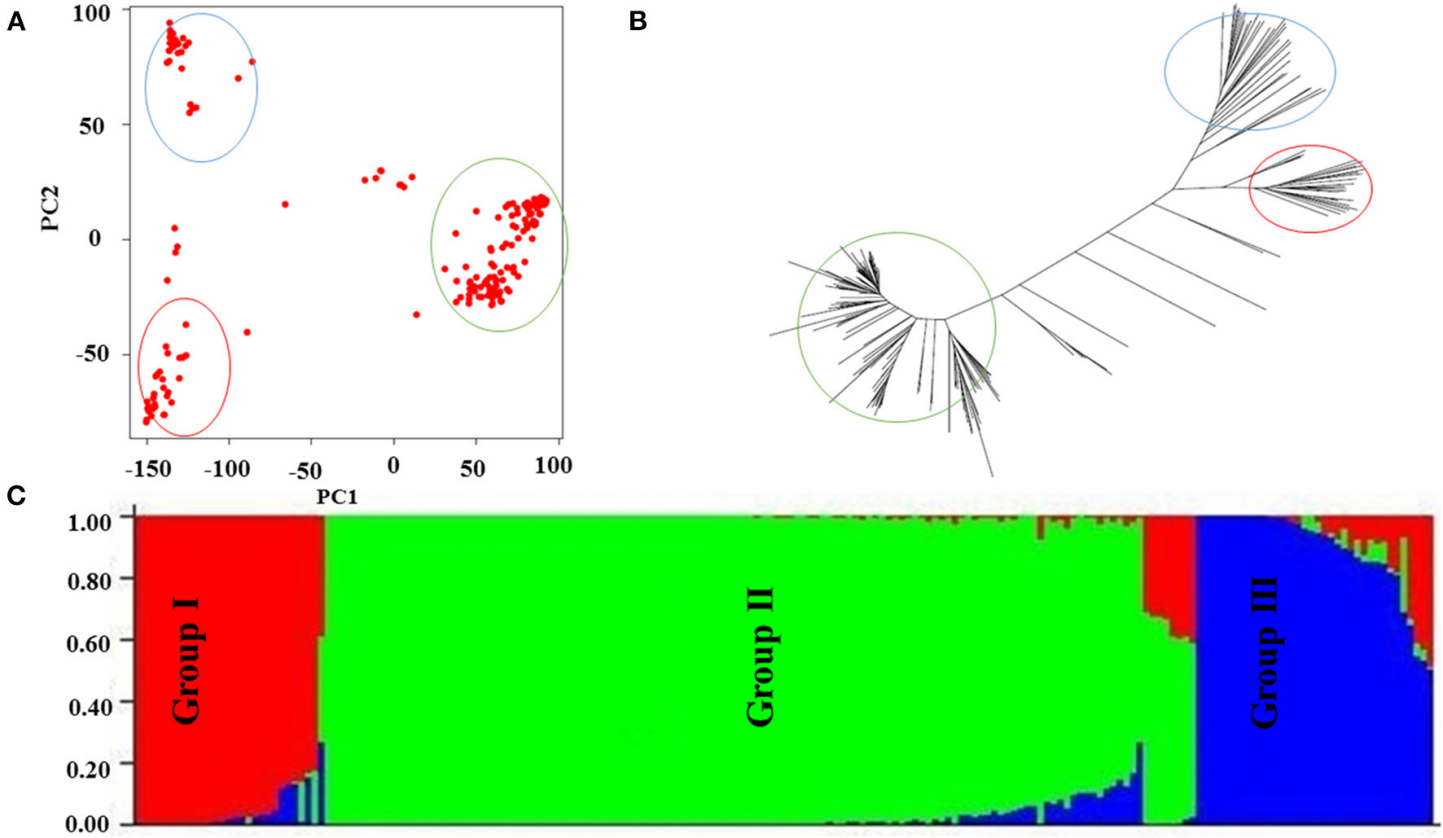
Population structure of current association panel. **(A)** Population structure of rice germplasm collection as reflected by principal components (PCs). The principal components analysis (PCA) plot of the first two components divided the rice panel into three main groups, **(B)** the dendrogram of neighbor-joining clustering, constructed using 36,901 SNPs, and the results show that genotypes are divided into three main groups, and **(C)** Bayesian clustering of 197 rice accessions using STRUCTURE; again, the results show that genotypes are divided into three main groups.

Genomic association and prediction integrated tool software was used to characterize the population structure, and three main groups were found (Figure 3A) as illustrated by the neighbor-joining dendrogram of all accessions (Figure 3B), reflecting the results from PCA. The PCA results from TASSEL showed that the first two PCs explained 8.2 and 7.2% of the variations, respectively, and are likely good enough for summarizing the population structure in the SNP data. STRUCTURE showed that the entire population consisted of three subgroups (Figure 3C).

### Analysis of LD and LD Decay

**Figure 4:**
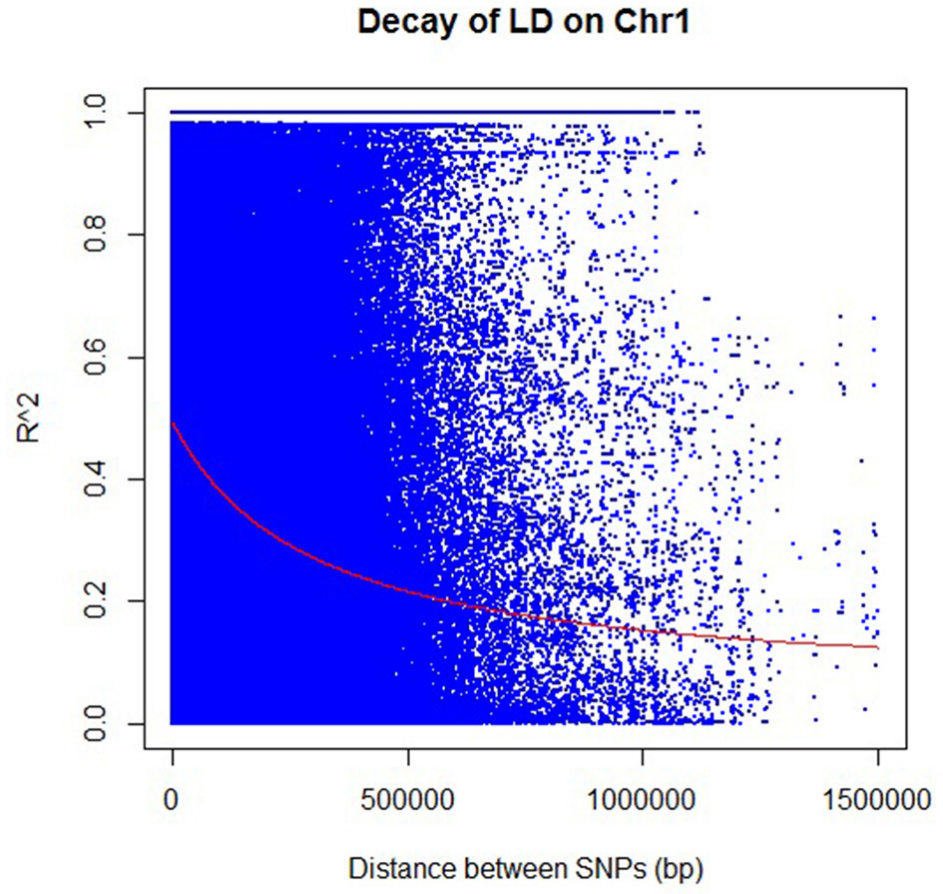
Rice chromosome 1 linkage disequilibrium (LD) decay as the representative figure for other chromosomes (plots for other 11 chromosomes are in Supplementary Figure 4). Each dot represents LD (*r*^2^) between a pair of SNPs, as a function of the distance (in bp). The red line shows the regression used to describe the relationship between the pairwise distance and *r*^2^.

The decay distances of the LD between SNPs were calculated using TASSEL and R software (Figure 4; Supplementary Figure 4). Altogether, 82.4% of the SNPs had significant LD (*p* < 0.01), and all chromosomes showed LD decay. The LD decay in the population was very slow, dropping to 0.2 in around ~570 kb (~2 cM) and 0.1 in around 1.65 Mbp (<5 cM) (Figure 4; Supplementary Table 2). Estimating the LD in the population proved to be consistent with other results in rice (Mather et al., 2007). The amount of LD based on *r*^2^ values varied from 0.232 for chromosome 4 to 0.531 for chromosome 5 with a mean value of 0.321 for all chromosomes. The distribution of knowledge points within the plot of LD (*r*^2^) decay against the distance (cM) within the 12 chromosomes showed that LD was not an easy monotonic function of the distance between markers. However, *r*^2^ decreased as the genetic distance between loci pairs increased, indicating that the probability of LD is low between distant loci pairs.

### Genome-Wide Association Study

**Figure 5:**
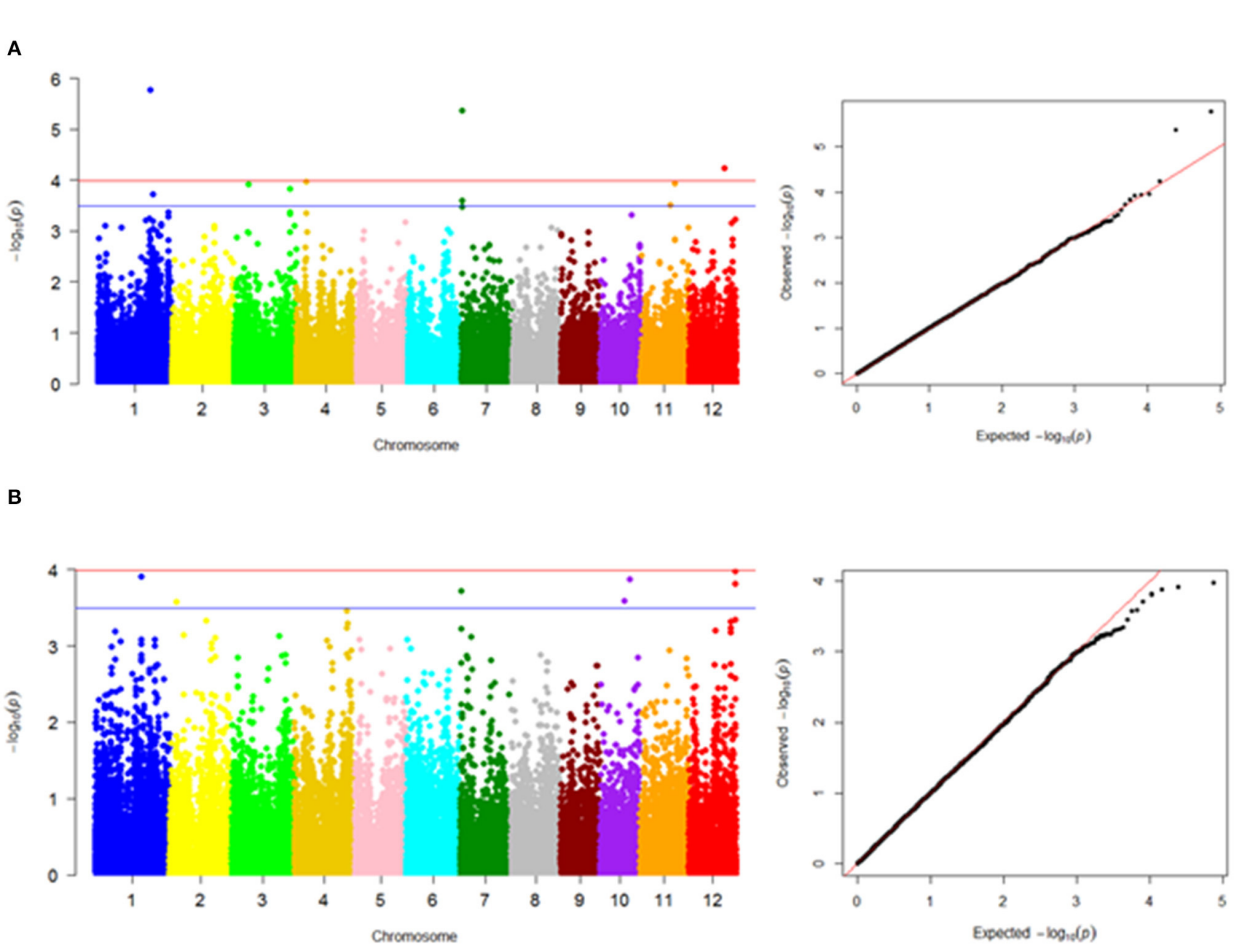
Manhattan plots showing marker-trait associations in genome-wide association study results using the Fixed and Random Model Circulating Probability Unification (FarmCPU) model. **(A)** MLG content and **(B)** starch content. The –log10(*p* values) on the left scale of Manhattan plots are plotted against the position of SNPs on each of the 12 rice chromosomes. The Quantile–quantile plot (on the right) illustrates the distribution of expected vs. observed probability values, represented based on –log_10_(*p*). For MLG, four loci (on chromosomes 1, 4, 7, and 12) and for starch, one locus (on chromosome 12) were identified above the Bonferroni threshold (red line) correcting for genome-wide multiple tests at a type I error of 0.0001. The blue line on each Manhattan plot indicates significant marker-trait associations with –log_10_(*p*) > 3.5.

The FarmCPU model in GAPIT software provided a stronger control of confounding effects when compared with MLM and GLM in TASSEL and MLM and MLMM in GAPIT (Supplementary Figure 5). The literature has shown that the statistical power of FarmCPU > MLMM > MLM > GLM (Lipka et al., 2012). The Q-Q plot results of the abovementioned models are presented in Supplementary Figure 5 for better comparison of these different models. The FarmCPU model resulted in more associations than expected and revealed 98 and 66 significant marker-trait associations with –log_10_(*p*) > 3.5 for MLG and starch contents, respectively (Supplementary Table 3); among the genes located in the vicinity of marked regions, we reported 9 and 5 genes as primarily candidate genes for MLG and starch contents, respectively (Table 2; Figure 5). Seven genes for MLG had an FDR <0.05 and, hence, can be considered as candidate genes. However, based on –log_10_(*p*) > 3.5, five genes were reported for the starch content, with regard to FDR selection, and only two genes passed the threshold of FDR <0.05; thus, hereafter, we do not rely on the other three genes.

**Table 2:** SNP association with MLG, starch, and biological function of associated genes.

**Trait**	**Marker**	**Chr**.	**QTL**	**Position (bp)**	**–Log_**10**_(*p*)**	**FDR*s***	**Associated genes**	**Biological process**	**Co-expressed gene list obtained by Genevestigator**
MLG	id1018389	1	qMLg1.1	30647512	5.8	0.0031	Anthocyanidin 5,3-O-glucosyltransferase	Detoxification	Sucrose-phosphate synthase
	id7000056	7	qMLg7.1	349201	5.4	0.0039	Hexose transporter	Hexose transformation	GH17, XG_FTase, glycosyltransferase
	id12006782	12	qMLg12.1	20828974	4.2	0.0354	Expressed protein	–	–
	id4002459	4	qMLg4.1	5931193	4.0	0.0371	OSIGBa0102N07	–	Glucosyltransferase, GH17
	id3004692	3	qMLg3.1	8841311	3.9	0.0371	Fructose-1,6-bisphosphatase	Sucrose biosynthetic	–
	id3015813	3	qMLg3.2	32757036	3.8	0.0394	Fasciclin protein	Cell adhesion	Glucan endo-1,3-beta-glucosidase, CESA1, CSLF6, GH16, GT, CESA6, CESA5, GH3
	id1019609	1	qMLg1.2	32145847	3.7	0.0486	X8 protein	Carbohydrate metabolic	Glucan endo-1,3-beta-glucosidase, GH16, beta-D-xylosidase
	id7000063	7	qMLg7.2	336771	3.6	0.0734	Ser/Thr protein phosphatase	Cell signaling	CslA9, CslC10
	id11006408	11	qMLg11.1	17911046	3.5	0.1145	Glycosyl transferase 31	Transferase activity	glycosyl hydrolase
Starch	id12010038	12	qSTh12.1	27330083	4.00	0.03219	Nodulin-like domain	Electron transfer activity	GH16
	id1015655	1	qSTh1.1	26874027	3.9	0.04000	Kelch repeat containing protein	Anaerobic respiration	–
	id7000089	7	qSTh7.1	630682	3.7	0.362676	WRKY 29	Transcription factor	UDP-glucoronosyl/UDP-glucosyl transferase
	id10003834	10	qSTh10.1	14516704	3.6	0.379737	Expressed protein	–	–
	id2002160	2	qSTh2.1	4038445	3.6	0.379737	DUF284	–	–

Significant SNPs identified in our GWAS analysis were searched against the rice annotation project database (https://rapdb.dna.affrc.go.jp/). In the vicinity of detected QTLs for MLG, the following candidate genes were identified in decreasing order of importance based on the corresponding *p*-values and FDRs: qMLg1.1 (anthocyanidin 5,3-*O*-glucosyltransferase), qMLg7.1 (hexose transporter and expressed protein), qMLg4.1 (OSIGBa0102N07 protein), qMLg3.1 (fructose-1,6-bisphosphatase), qMLg3.2 (fasciclin domain-containing protein), qMLg1.2 (X8 domain-containing protein), qMLg7.2 (Serine and threonine (Ser/Thr) protein phosphatase family protein), and qMLg11.1 [glycosyltransferase family 31 enzymes (GT31)] (Figure 5A, Table 2). Also, Bonferroni correction confirmed the first four QTLs on chromosomes 1, 7, 12, and 4 (including qMLg1.1, qMLg7.1, qMLg12.1, and qMLg4.1) to be above the threshold correcting for genome-wide multiple tests at type I error of 0.0001 (Figure 5A). In the vicinity of QTLs for the starch content, the following candidate genes were identified: qSTh12.1 (nodulin-like domain) and qSTh1.1 (kelch repeat-containing protein) (Figure 5B, Table 2).

The results of co-expressed genes using Genevestigator for the associated genes with SNP markers for both MLG and starch contents are presented in Table 2 and discussed herein. The physical locations of these QTLs are shown in Supplementary Figure 6.

### Expression Assay of Candidate Genes

The expression analysis by RGAP (http://rice.plantbiology.msu.edu/) revealed the differential expression of most of the candidate genes in different tissues by RNA-Seq assay (Supplementary Table 4), which was cross-validated by TRAP-Seq assay. The genes LOC_Os01g53330 and LOC_Os03g16050, both associated with MLG, showed the maximum expression level in seedlings, and the genes LOC_Os07g01560, LOC_Os11g30760 (associated with the MLG), and LOC_Os01g47050 (associated with the starch content) showed the maximum expression level in callus, whereas the genes LOC_Os03g57460 and LOC_Os01g55820, both associated with MLG, showed the maximum expression level in panicles. The network analysis using the RiceFREND database showed the co-expression pattern of the candidate genes and interaction in gene networks. For each candidate gene, 2–6 direct interactions were detected in gene networks (Supplementary Table 5). For example, in the case of LOC_Os07g01560 (hexose transporter) which is associated with MLG, it is co-expressed with four genes, namely, LOC_Os04g52190 (similar to vacuolar sorting receptor 7), LOC_Os04g37980 (monosaccharide transporter 1), LOC_Os01g72170 (glutathione *S*-transferase GST 29), and LOC_Os03g11900 (hexose transporter).

The analysis of the candidate genes using KEGG (https://www.kegg.jp/) revealed three genes associated with MLG with a KEGG identifier (Supplementary Table 6), namely, LOC_Os07g01560, LOC_Os03g16050, and LOC_Os12g34360 with KO numbers K24193, K03841, and K19023, respectively. The KEGG pathway for the first gene was osa02000 (transporters, sugar transporters, and sugar porter family); for the second gene, many KEGG pathways, such as osa00010 (glycolysis/gluconeogenesis), osa00030 (pentose phosphate pathway), osa00051 (fructose and mannose metabolism), osa00710 (carbon fixation in photosynthetic organisms), osa01100 (metabolic pathways), osa01110 (biosynthesis of secondary metabolites), and osa01200 (carbon metabolism), were identified; for the third gene, one KEGG pathway was identified as osa03400 (double-strand breaks repair [DSBR] and homologous recombination [HR]). The GO analysis using PANTHER (http://pantherdb.org/) classified the candidate genes in view of GO terms into the cellular component (CC), molecular function (MF), and biological process (BP). For example, LOC_Os03g16050 (fructose-1,6-bisphosphatase), which is associated with MLG, has GO identifiers of CC: cytosol (GO:0005829), MF: phosphatase activity (GO: 0016791), and BP: cellular carbohydrate biosynthetic process (GO:0034637), organophosphate metabolic process (GO:0019637), phosphate-containing compound metabolic process (GO:0006796), cellular carbohydrate biosynthetic process (GO:0034637), oligosaccharide biosynthetic process (GO:0009312), and glucose metabolic process (GO:0006006) (Supplementary Table 6).

## Discussion

Cereals are an important source of dietary carbohydrates. MLGs are important components of the grain endosperm cell wall and constitute a large fraction of the fiber content that is known to have beneficial effects on human health. The high importance of these carbohydrates should render them a focus of plant breeding to improve the nutritional quality of rice, but few studies have so far assessed the diversity of the MLG content and its genetic basis in rice. To the best of our knowledge, this is the first GWAS report for the MLG content in rice grains. The rice accessions showed a considerable variation in the MLG content in the whole grain [coefficient of variation (CV) = 37.1%] (Table 1); the MLG content ranged between 27 and 398 μg/mg of whole grain with an average of 252 μg/mg, that is, MLG constitutes 2.7–39.8% of rice whole grain. It must be noted that the amount of this carbohydrate in rice whole grain has not yet been measured, but its content was measured in the endosperm of different cereals including rice. However, the reported values are very inconsistent; so that 0.4–0.9% (Demirbas, 2005), <0.06% (Burton and Fincher, 2012), and up to 20% (Fincher and Stone, 2004) were reported in rice endosperm. The MLG content was reported to account for 20% of the total dietary fiber content in wheat (Prasadi and Joye, 2020). Wirkijowska et al. (2012) reported that the content of (1–3) (1–4)-β-d-glucans in barley kernel ranged between 4.04 and 5.71% of dry matter, and Garcia-Gimenez et al. (2019) expressed that barley has a considerably higher MLG content (4–10%) compared with wheat (1%) or rice (<0.06%), although (Fincher, 2009a) reported that (1–3) (1–4)-β-d-glucans constitute up to 70% of starchy endosperm in barley. In all these reports, the amount of MLG was measured in a few samples/accessions. In the case of the starch content, the accession panel showed a relatively high variation (CV = 21.2%) (Table 1). The starch content in whole grain ranged between 321 and 869 μg/mg with an average of 561 μg/mg, that is, starch constitutes 32.1–86.9% of rice whole grain. As reported by Moongngarm (2013), rice endosperm contains up to 70% of starch, which is not found in the cell wall itself. In other studies using a few samples, the starch content of rice milled grain was reported between 78.1 and 85.6% (Fujita et al., 2007) and 80 and 82% (Kim et al., 2021).

Most of the GWAS mapping studies in rice so far have been conducted to identify genes or QTLs related to agronomic performance such as yield and grain quality traits (Borba et al., 2010; Courtois et al., 2013; Zhang et al., 2014; Begum et al., 2015; Fei-fei et al., 2018), resistance to abiotic stresses (Famoso et al., 2011; Kumar et al., 2015; Swamy et al., 2017), eating quality (Zhao et al., 2013; Wang et al., 2017), and starch content (Yang et al., 2014; Bao et al., 2017). The correlation between starch and MLG contents was significantly negative, as reported previously (Harasym and Oledzki, 2018), following the extensive quantification of starch and MLG contents in our rice accessions. Using these quantitative experimental data, we have performed a GWAS on MLG and starch contents, looking at 33,812 SNPs across the entire genome of rice. The population structure and kinship (K) matrices were used to reduce spurious associations. Genes we identified as being associated with MLG and starch contents may contribute to their natural variation in rice accessions. Many genes other than the expected *CslF6* and *CslH* are associated with the MLG content (Hazen et al., 2002; Houston et al., 2014) and may play a role in controlling the biosynthesis or biodegradation of MLG; the discovery of an association between *CslC, XTH*, and starch content supports these observations.

We selected and used a FarmCPU model for association mapping based on the comparison of different models. In fact, in the GLM, the population structure may be used as a cofactor to partially account for the residuals and reduce the false positives *via* the removal of the effects that are independent of the marker effect. The MLM provides the opportunity to define interrelationships among individuals that can be calculated in the variance-covariance matrix of individuals presented as K matrix and/or the analysis of population structure known as “*Q*” matrix. The latter can be studied *via* STRUCTURE (Pritchard et al., 2000) or principal component analysis (PCA; Zhao et al., 2007). In cases where both *Q* and *K* are being implemented in the analysis of individuals, the power of statistics is more reliable than using either case alone (Yu et al., 2006). The other common model that takes an advantage of forward-backward stepwise linear mixed-model regression is MLMM, which uses associated markers as covariates (Lipka et al., 2012). More recently, to control the false positives and minimize the problem of the confounding effect of markers and cofactors at once, FarmCPU (as used in this study) was developed that additionally solves over model fitting problem *via* considering the associated markers to be random (Liu et al., 2016).

### Candidate Genes for MLG

The heritability measure for MLG in the rice panel was 0.87, which is consistent with what has been reported for other grasses (Cervantes-Martinez et al., 2001; Kim et al., 2011; Marcotuli et al., 2016). GWAS analyses showed seven candidate genes to be associated with the MLG content in rice and these are discussed as follows in terms of their potential involvement in MLG biosynthesis or the regulation thereof.

#### *Anthocyanidin 5,3-O-*Glucosyltransferase

The PANTHER analysis revealed that the gene belongs to the family/subfamily of GLYCOSYLTRANSFERASE/GLYCOSYLTRANSFERASE (PTHR48048:SF27) (Supplementary Table 6). The enzyme (UA3GT) transfers UDP-glucose to the aglycone (anthocyanidin) to make 3-mono-*O*-glycopyranosyl anthocyanin (anthocyanidin 3-glycoside), one of the simplest anthocyanins. Our co-expression analyses by using Genevestigator showed that UA3GT co-expresses with sucrose-phosphate synthase (Table 2). Sucrose-phosphate synthase contributes to the synthesis of photosynthetic sucrose by interfering with the rate-limiting step of sucrose biosynthesis from UDP-glucose and fructose-6-phosphate (Stein and Granot, 2019). Although no direct relationship with MLG has been established for UA3GT, it would be valuable to investigate the content and structure of β-glucans in UA3GT loss-of-function mutants.

#### *Hexose* Transporter

Hexose transporters, a large family of monosaccharide transporters, are involved in proper carbon portioning within plants (Buttner and Sauer, 2000). The KEGG analysis revealed that the gene belongs to pathway osa02000 (transporters, sugar transporters, and sugar porter (SP) family), and the PANTHER analysis showed that it belongs to the protein class of secondary carrier transporter (PC00258) (Supplementary Table 6). The co-expression analyses by using Genevestigator showed that the hexose transporter highlighted in this study co-expresses with β*-1,3-glucanosyltransglycosylase* (GH17) and XG FUT (GT37) (Table 2). The GH17 family of hydrolases includes enzymes capable of deconstructing MLG (Woodward and Fincher, 1982; Houston et al., 2014), which would have a clear role in MLG metabolism. The expression of a putative XyG biosynthetic gene suggests that the *O*. *sativa* cell wall, thought to contain only barely detectable levels of XyG with no additional glycosyl substitutions, could be reinvestigated to check the possibility of further substitution of fucosylated XyG. The latter suggests the bioavailability of minute amounts of XyG in rice grain, making a minor contribution to the overall MLG content (Perrin et al., 1999). As reported earlier, the xylosyl substituent of XyG is not being further substituted with other aglycons in rice (Kato et al., 1982; Figure 2B).

#### *Fructose-1,6-*Bisphosphatase

As per the KEGG analysis, this enzyme plays a role in glycolysis/gluconeogenesis (osa00010) and fructose and mannose metabolism (osa00051). Based on the GO analysis using PANTHER, the enzyme is targeted to the cytosol (GO: 0005829) to perform its phosphatase activity (GO: 0016791) in BPs including oligosaccharide biosynthetic process (GO: 0009312) and glucose metabolic process (GO: 0006006) (Supplementary Table 6). So far, no study suggests any relation of fructose to the MLG content, and so this association needs to be further investigated *via* functional analysis. Furthermore, the co-expression analysis did reveal any tangible relation to other cell wall-related genes.

#### *Fasciclin* Domain-Containing Protein

Although KEGG analysis failed to identify its pathway (Supplementary Table 6), the structural fasciclin domain-containing proteins belong to a subgroup of arabinogalactan-proteins (AGPs) and have AGP-like glycosylated regions known as fasciclin domains (Johnson et al., 2003). The relevance of these proteins to wheat flour milling has been demonstrated (Nirmal et al., 2017(. In rice, family members of 27 fasciclin-like arabinogalactan (FLA) proteins were identified (Faik et al., 2006). In cotton, an FLA gene was reported to be related to the synthesis of the primary cell wall: the overexpression of GhFLA1 increased the amount of pectin and decreased the amounts of hemicellulose and cellulose (Huang et al., 2013). The results of the co-expression analysis showed that this gene co-expresses with cell wall-related genes including *glucan endo-1,3-beta-glucosidase, CESA1, CSLF6, GH16, CESA6, CSLA1*, and *CESA5*.

#### *X8* Protein

In the KEGG analysis, this protein was defined as plasmodesmata callose-binding protein 3 which belongs to the BETA-1,3 GLUCANASE protein family (Supplementary Table 6). The X8 module is found at the COOH-terminus of GH17 β-1,3-glucanases and contributes to β-1,3-glucan-binding (Henrissat and Davies, 2000). The co-expression analyses showed that the X8 protein co-expresses with *glucan endo-1,3-beta-glucosidase* and *glycosyl hydrolases* of GH17, which are pivotal enzymes involved in MLG hydrolysis during germination (Varghese et al., 1994; Millet et al., 2018).

#### *Serine/*Threonine-Protein Phosphatase Family Protein

Ser and Thr are the most frequently phosphorylated residues in eukaryotic proteins; the phosphate group can be removed from these amino acids by Ser/Thr protein phosphatases (Hardie, 2000). The activity of these enzymes has an enormous impact on cellular functions like stress signaling, cell integrity, tip cell growth, regulation of metabolism, cell cycle and development, in addition to roles in response to light and stress, and hormonal signaling (Afzal et al., 2008). So far, there is no indication of any involvement of these enzymes with the synthesis or degradation of MLG or other cell wall components. However, the data demonstrate that the gene co-expresses with *CslA9* and *CslC10*, prompting a diligent future look to this family of regulatory proteins and their interrelationship with cell wall components, including mannans and XyGs, the likely products of *CslA9* and *CslC10* enzymes.

#### *Glycosyl* Transferase 31 (GT31)

The involvement of many GT families, including GT2, GT8, GT31, GT34, GT37, and GT47s, in cell wall biosynthesis, has been demonstrated, as summarized in https://cellwall.genomics.purdue.edu/families/index.html. Enzymes from family GT31 have various functions including β-1,3-galactosyltransferase, fucose-specific β-1,3-*N*-acetylglucosaminyltransferase, and globotriosylceramide synthase (Strasser et al., 2007). Members of GT31 have been hypothesized to be involved in cell wall biosynthesis (Cao et al., 2008; Bosch et al., 2011). The rice genome encodes 10 GT31 enzymes (Paterson et al., 2009), most of which are expressed during primary wall formation. Recently, it was reported that GT31 family members are predicted to form the (1→3)-β- and (1→6)-β-linked galactan chains of type II AGPs. Also, studies showed that the GT31 genes are expressed during primary cell wall formation, and the expressions of *GT31A3* (member of GT31 subgroup A) and *GT31F4* and *GT31F5* (two members of subgroup F) mainly affect secondary cell wall formation. (Penning et al., 2019).

### Candidate Genes for Starch

The heritability of starch was 0.9 as reported previously (Rathi et al., 2010). GWAS analyses showed only two candidate genes with possible relations to the starch content, as discussed below.

#### *Nodulin-Like* Domain-Containing Protein

Although the KEGG analysis did not show its undelaying pathway, the GO analysis using PANTHER revealed that this protein is targeted to the membrane (GO:0016020) with any known MF or BP (Supplementary Table 6). However, based on the literature, these proteins are involved in the catabolism of sucrose and starch (Dixon, 1984). Khan et al. (2007) showed that mutations in this gene in *Arabidopsis* reduced the amount of starch present in tissues. To the best of our knowledge, this is the first report of a relatively high association of nodulin-like protein to the starch content in monocots and suggests that further functional analysis is required.

#### *Kelch* Repeat-Containing Protein

This protein is encoded by *OsFBK1* and belongs to the protein class of ubiquitin-protein ligase (PC00234). It is targeted to SCF ubiquitin ligase complex (GO: 0019005) to be involved in the SCF-dependent proteasomal ubiquitin-dependent protein catabolic process (GO: 0031146) (Supplementary Table 6). The *OsFBK1* functions in proteolysis, and a recent study showed that it affects the secondary cell wall thickenings of anthers and roots by regulating the level of Cinnamomyl-CoA Reductase (CCR), which controls lignification in rice (Borah and Khurana, 2018). This gene also plays a significant role in acquiring drought tolerance in rice (Borah et al., 2017). Kelch proteins have substantial roles in the circadian clock and flowering time regulation (Naeem ul Hassan et al., 2015). Sulpice et al. (2009) demonstrated a correlation of the corresponding transcripts with biomass production and starch content in *Arabidopsis*. As with the nodulin-like protein, this is the first report on the association of this gene in monocots with the starch content.

## Conclusion

In this study, the MLG and starch contents of rice whole grains were measured in a large portfolio of rice accessions. Using these biochemical data, a GWAS was performed on 197 rice accessions, comparing MLG and starch contents with genomic SNP profiles. This study identified seven significant QTLs associated with the MLG content and two QTLs associated with the starch content in rice whole grain, respectively, *via* GWAS in 197 rice accessions. The present findings provide useful information for selecting candidate genes and may be helpful to molecular breeding. The amount of both MLG and starch contents in rice grains is of economic importance and is relevant due to the inherent nutritional significance. Herein, genes with putative association MLG and starch contents in rice whole grain are newly reported. The findings propose future functional genetic studies, as the precise involvement of most of the identified associated genes is unknown; however, we obtained the results of the expression analysis of candidate genes by using RNA-Seq and TRAP-Seq assays from a public annotation project, RGAP (http://rice.plantbiology.msu.edu/) to have a general view of gene action in different tissues of rice (i.e., seedling, callus, and panicle) (Supplementary Table 4). Defining the mechanistic involvement of these apparently associated genes will be potentially game-changing in the area of food nutrition and in feed industries that are reliant on starch and cell wall polysaccharides. Furthermore, the data provide insight that will be useful in the design of future breeding programs, allowing breeders to use available genetic resources more effectively in meeting global food demand and supply.

## Data Availability Statement

The original contributions presented in the study are included in the article/Supplementary Material, further inquiries can be directed to the corresponding author/s.

## Author Contributions

RP conducted the research as her PhD dissertation. AA proposed the research idea, conducted some analyses, and supervised all issues of the research. LM conducted some laboratory evaluations. PI conducted some laboratory evaluations and helped GWAS analysis. NF co-supervised the research and conducted some analyses. All authors contributed to the article and approved the submitted version.

## Conflict of Interest

The authors declare that the research was conducted in the absence of any commercial or financial relationships that could be construed as a potential conflict of interest.

## Publisher's Note

All claims expressed in this article are solely those of the authors and do not necessarily represent those of their affiliated organizations, or those of the publisher, the editors and the reviewers. Any product that may be evaluated in this article, or claim that may be made by its manufacturer, is not guaranteed or endorsed by the publisher.
